# Compassionate use of contezolid in a toddler with severe community-acquired pneumonia induced by staphylococcus aureus: a case report and follow-up

**DOI:** 10.3389/fped.2024.1321447

**Published:** 2024-02-07

**Authors:** Hui-Ying Liu, Xiao-Fei Bi, Ya-Jun Wang, Feng-Jie Xie, Hong Zhang, Yu-Cheng Zhu, Yan Zhang, Zhi-Hui Wang, Di Wu, Huan Meng, Yi-Lu Lin, Lin-Qiong Liu, Shu-Xiao Qiu, Yan Gao, Kai Kang, Yang Gao

**Affiliations:** ^1^ Department of Critical Care Medicine, The Sixth Affiliated Hospital of Harbin Medical University, Harbin, Heilongjiang, China; ^2^ Department of Pediatrics, The Sixth Affiliated Hospital of Harbin Medical University, Harbin, Heilongjiang, China; ^3^ Department of Critical Care Medicine, The Hongqi Hospital Affiliated to Mudanjiang Medical University, Mudanjiang, Heilongjiang, China; ^4^ Department of Critical Care Medicine, The Hongxinglong Hospital of Beidahuang Group, Shuangyashan, Heilongjiang, China; ^5^ Department of Emergency, The Sixth Affiliated Hospital of Harbin Medical University, Harbin, Heilongjiang, China; ^6^ Department of Critical Care Medicine, The Fourth Affiliated Hospital of Harbin Medical University, Harbin, Heilongjiang, China; ^7^ Department of Emergency, The Fourth Affiliated Hospital of Harbin Medical University, Harbin, Heilongjiang, China; ^8^ Department of Critical Care Medicine, The First Affiliated Hospital of Harbin Medical University, Harbin, Heilongjiang, China

**Keywords:** Contezolid, Compassionate use, Toddler, Community-acquired pneumonia, Staphylococcus aureus, Gram-positive bacterial infection, Adverse drug reactions

## Abstract

**Background:**

Initial choices of antimicrobial therapy for most cases of community-acquired pneumonia (CAP) in children under 5 years of age are typically based on local epidemiology, risk factors assessment, and subsequent clinical parameters and positive cultures, which can lead to the underdiagnosis and underestimation of lung infections caused by uncommon pathogens. Contezolid, an orally administered oxazolidinone antibiotic, gained approval from the National Medical Products Administration (NMPA) of China in June 2021 for managing complicated skin and soft tissue infections (cSSTI) caused by staphylococcus aureus (SA), streptococcus pyogenes, or streptococcus agalactis. Owing to its enhanced safety profile and ongoing clinical progress, the scope of contezolid's clinical application continues to expand, benefiting a growing number of patients with Gram-positive bacterial infections.

**Case summary:**

In this report, we present the first use of contezolid in a toddler with severe CAP caused by SA, aiming to avoid potential adverse drug reactions (ADRs) associated with vancomycin and linezolid.

**Conclusion:**

Although contezolid has not been officially indicated for CAP, it has been shown to be effective and safe in the management of SA-induced severe CAP in this toddler, suggesting its potential as an alternative option in the dilemma, especially for patients who are susceptible or intolerant to ADRs associated with first-line anti-methicillin-resistant staphylococcus aureus (MRSA) antimicrobial agents.

## Introduction

Community-acquired pneumonia (CAP) is most prevalent among children under 5 years old (1). Identifying CAP typically involves a thorough assessment of the patient's medical history, clinical symptoms, physical examination, laboratory tests, and imaging results (2). In children under 5 years old, the most commonly identified bacteria causing CAP include streptococcus pneumoniae, streptococcus pyogenes, klebsiella pneumoniae, escherichia coli, staphylococcus aureus (SA), haemophilus influenzae, haemophilus parainfluenzae, and moraxella catarrhalis (3, 4). The presence of significant pleural effusion or extensive multi-lobar infiltrates or consolidations on lung imaging is often indicative of necessity of hospitalization, disease severity, need for intensive care unit (ICU)-level care and organ function support, and a less favorable prognosis (5, 6). Prompt diagnosis, pathogen identification, risk stratification, and subsequent targeted antimicrobial therapy are vital for favorable outcomes in children with CAP (7). In clinical practice, initial choices of antimicrobial therapy for most cases of CAP in children under 5 years of age are typically based on local epidemiology, risk factors assessment, and subsequent clinical parameters and culture results, which can lead to the underdiagnosis and underestimation of lung infections caused by uncommon pathogens (8). By amplifying and sequencing the nucleic acids of isolated pathogens, metagenomic next-generation sequencing (mNGS) can offer superior performance in identifying pathogens from various body parts with higher sensitivity and diagnostic specificity than traditional pathogen detection methods, particularly for rare pathogens (9, 10).

Although not the most common, staphylococcus aureus (SA), including the methicillin-resistant SA (MRSA) variant, does play a role as one of the pathogens responsible for pediatric CAP, which can be partly attributed to its natural colonization in the airways (11, 12). More importantly, SA-induced CAP is associated with unfavorable clinical outcomes compared to the common pneumococcal CAP, involving a higher risk for sepsis and septic shock, need for ICU-level care and organ function support, and significant mortality, regardless of the presence of methicillin-resistance (13, 14). However, the current practice of empirical antimicrobial agents for MRSA-induced lung infections remains suboptimal in consideration of potential adverse drug reactions (ADRs) associated with first-line anti-MRSA antimicrobial agents, leading clinicians have to carefully weigh the pros and cons of antibiotic choices (15, 16). This predicament underscores the pressing need for the development of new antibiotics, leading to the emergence of contezolid. Conteziold, an orally administered oxazolidinone antibiotic, gained approval from the National Medical Products Administration (NMPA) of China in June 2021 for managing complicated skin and soft tissue infections (cSSTI) caused by SA, streptococcus pyogenes, or streptococcus agalactis (17). Owing to its enhanced safety profile and ongoing clinical progress, the scope of contezolid's clinical application continues to expand, benefiting a growing number of patients with Gram-positive bacterial infections (18–21).

Notably, there have been no reports of the clinical application of contezolid in treating severe CAP caused by SA in the toddler. In this report, we present, for the first time, the compassionate use of contezolid in the treatment of severe CAP induced by SA in a toddler, demonstrating its favorable therapeutic efficacy and enhanced safety profile.

## Case presentations

### Chief complaints

A 28-month-old girl was admitted to the Department of Critical Care Medicine at the Sixth Affiliated Hospital of Harbin Medical University on September 26, 2022, due to a one-day history of fever and recurrent convulsions.

### History of present illness

The day before admission, the toddler had intermittent fever, with her body temperature reaching as high as 38.8°C at night, which could be temporarily relieved through physical cooling and oral antipyretic medication. In the early morning of admission, the toddler experienced another episode of fever accompanied by a convulsion, manifested as loss of consciousness, unresponsiveness to calls, cyanosis of the lips, clenching of the teeth, clenched fists, flexion of the upper limbs, stiffness of the lower limbs, and urinary and fecal incontinence, which lasted for a few minutes and resolved spontaneously. A similar convulsion recurred half an hour later and did not improve after receiving chloral hydrate enema and intravenous dexamethasone at the local hospital.

### History of past illness

The toddler had no previous history of illness.

### Personal and family history

There was no relevant personal or family history.

### Physical examination

Upon admission, the physical examination revealed the following parameters: height 0.86 meters, weight 12 kg, body mass index (BMI) 16.22 kg/m^2^, body temperature 36.0°C, heart rate (HR) 230 beats/min, blood pressure (BP) 96/60 mmHg, respiratory rate (RR) 83 breaths/min, pulse oxygen saturation (SO2) 90%, and children Glasgow Coma Scale (GCS) score of 8 points. Additionally, coma, pharyngeal congestion, mild edema of bilateral bulbar conjunctiva, mild cyanosis of the lips, shortness of breath, three depressions sign, rough breathing sounds, and audible moist rales in both lungs were also observed.

### Laboratory parameters

**Figure 1:**
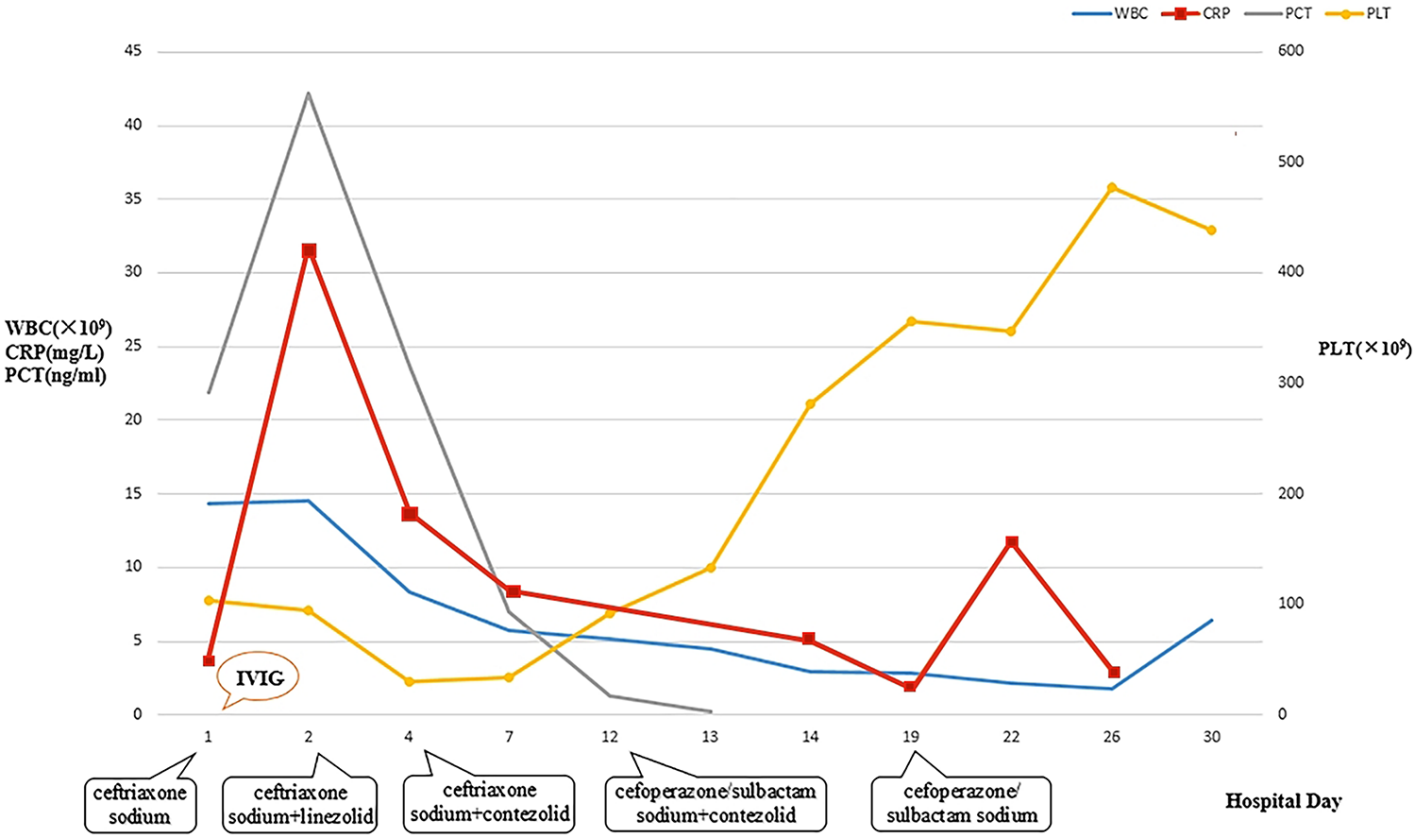
Dynamic changes of WBC, CRP, PCT, and PLT during hospitalization. WBC, white blood cell count; CRP, C-reactive protein; PCT, procalcitonin; PLT, platelet.

**Figure 2:**
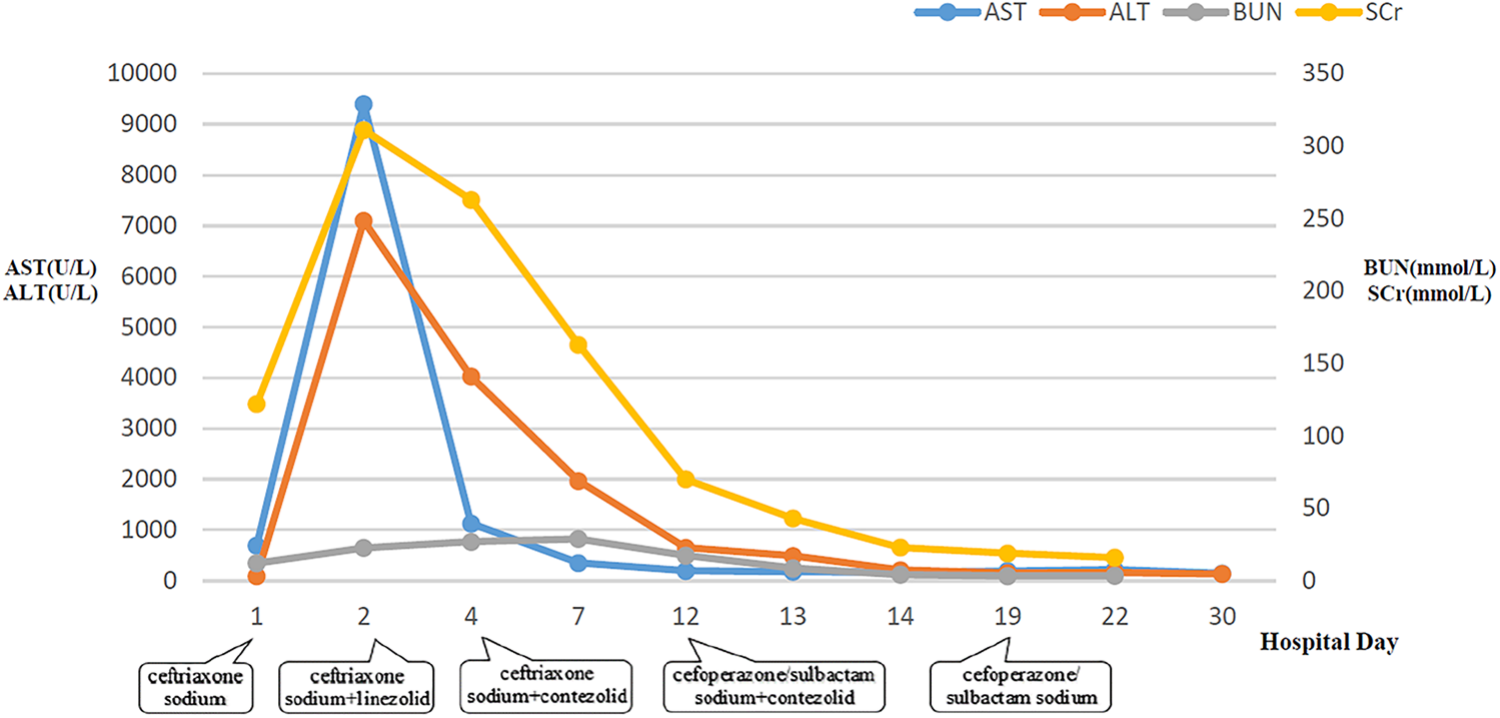
Dynamic changes of AST, ALT, BUN, and SCr during hospitalization. AST, aspartate aminotransferase; ALT, alanine aminotransferase; BUN, blood urea nitrogen; SCr, serum creatinine.

**Figure 3:**
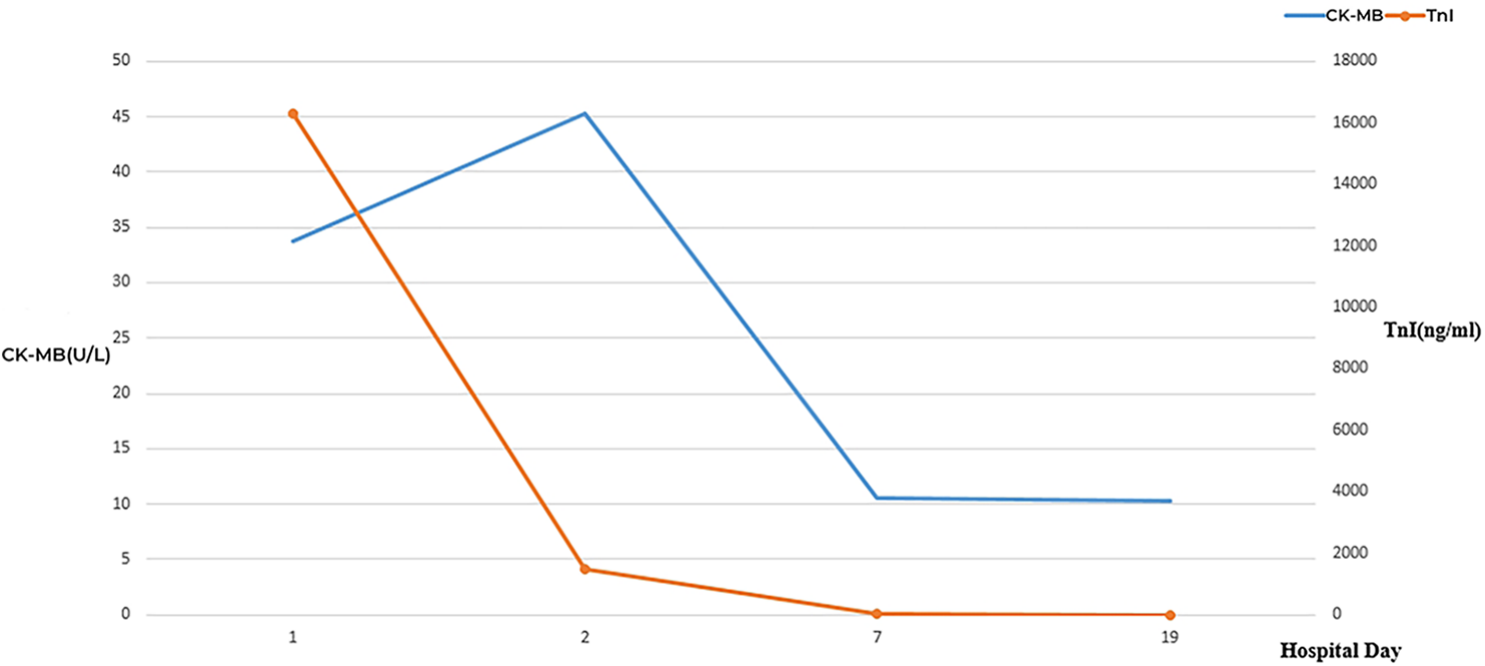
Dynamic changes of CK-MB and TnI during hospitalization. CK-MB, creatine kinase isoenzymes; TnI, troponin I.

The laboratory parameters upon admission and subsequent re-examinations during hospitalization are presented in Table 1 and Figures 1–3. Arterial blood gas analysis (ABGA) indicated the following abnormal values: pH 7.264, carbon dioxide partial pressure (PCO2) 27.0 mmHg, lactic acid (LAC) 10.3 mmol/L, and bicarbonate (HCO3-) 14.3 mmol/L. Occult blood was detected in gastric juice and stool samples. The Pediatric Critical Illness Score (PCIS) upon admission was 74 points. Nucleic acid tests for severe acute respiratory syndrome coronavirus type 2 (SARS-CoV-2), influenza A and B viruses, respiratory syncytial virus (RSV), as well as serological tests for mycoplasma pneumoniae (MP) and antibodies against various hepatitis viruses, syphilis, and human immunodeficiency virus (HIV) were all negative. During hospitalization, the results of multiple blood cultures were negative.

**Table 1:** Timeline of disease progression (September 26 to October 25, 2022).

Day of illness	1	2	4	7	12	13	14	19	22	26	30
Disease course	Hospitalization/ICU								Pediatric ward		Hospital discharge
WBC (×10⁹/L)	14.37	14.52	8.32	5.72	5.15	4.5	2.93	2.87	2.15	1.82	6.38
NEUT% (%)	35.5	96.6	91.7	86.3	75.1	73.7	66.4	55.5	28.2	53.8	53.3
NEUT (×10⁹/L)	5.11	14.02	7.64	4.93	3.88	3.32	1.95	1.6	0.61	0.98	3.4
LYM% (%)	62.4	1.6	3.8	4.7	18.7	16.6	24.1	28.2	42.6	29.1	23.8
LYMPH (×10⁹/L)	8.96	0.24	0.31	0.27	0.96	0.75	0.70	0.81	0.92	0.53	1.52
HGB (g/L)	118	115	99	97	98	93	105	104	96	92	95
HCT (%)	34.1	33.5	28.6	28.1	29.1	28.0	31.3	31.0	29.3	27.8	28.4
PLT (×10⁹/L)	104	94	30	34	92	133	282	356	347	477	438
PT (s)	51.3	>70	18.1	13.8	13.9	12.7		11.6			
PTA (%)	12.2	5.9	43.4	60.6	60.2	66.4		89			
INR	4.6	8.52	1.59	1.2	1.21	1.11		1.01			
APTT (s)	63.3	76.3	37.2	39.6	28.9	29.2		30.1			
FIB (g/L)	0.5	0.91	1.6	1.53	1.54	2.32		3.62			
TT (s)	40.8	124.5	25.7	34	19.5	20.4		24			
DD (g/ml)	>80	>80	36.54	9.81	11.28	8.43		2.49			
CK-MB (U/L)	33.7	45.3	106	10.6			8.79	10.3	11.3		
TnI (ng/ml)	16,320.1	1,479		55.99	34.42			0.016		<1.5	
NT-proBNP (pg/ml)	2,600	11,800						44.14		<10	
AST (U/L)	697	9,440	1,130	352	198	179	166	192	220		147
ALT (U/L)	91	7,100	4,028	1,968	651	492	211	152	163		134
ALB (g/L)	34	23.5	30.9	36.2	41.5	44	40.8	43.4	43		39.9
TBIL (mmol/L)	4.87	23.04	27.52	19.28	13.98	11.85	9.53	7.16	6.68		5.77
DBIL (mmol/L)	1.88	15.1	16.11	8.57	4.73	3.39	2.8	1.63	1.72		0.96
IBIL (mmol/L)	2.99	7.94	11.41	10.71	9.25	8.46	6.73	5.53	4.96		4.81
BUN (mmol/L)	12.23	22.69	26.98	28.91	17.47	8.72	4.24	3.36	3.46		
SCr (mmol/L)	122	311	263	163	70	43	23	19	16		
CRP (mg/L)	3.83	31.78	13.72	8.14			4.96	1.8	11.8	2.98	
PCT (ng/ml)	21.90	42.18	23.70	6.98	1.26	0.23		0.19			
Oxygen therapy	IMV				mask oxygen inhalation	tracheotomy + artificial nose oxygen inhalation					
PEEP (cmH₂O)	8	6	4	4							
FiO₂	80%	60%	40%	35%	29%	29%	29%	29%	25%	21%	21%
pH	7.294	7.312	7.326	7.359	7.41	7.282	7.399	7.466	7.426	7.43	7.428
PCO₂ (mmHg)	18.3	19.7	30.2	35.9	33.9	45.9	36.7	40.5	41.6	40.9	43.4
PO₂ (mmHg)	68.5	102	84	114	143	80.8	111	130	113	89.8	112
OI (mmHg)	85.6	170	210	325.7	493.1	278.6	382.8	448.3	452	427.6	533.3
LAC (mmol/L)	10.3	5.6	3.0	1.6	1.1	0.6	1.1	0.9	0.6	0.9	0.9
HCO₃ˉ (mmol/L)	13.2	13.3	17.1	20.5	22.2	20.3	22.9	29	26.9	27.5	27.9
Antibiotics	ceftriaxone sodium	ceftriaxone sodium + linezolid	ceftriaxone sodium + contezolid		cefoperazone/sulbactam sodium + contezolid			cefoperazone/sulbactam sodium			

### Imaging findings

**Figure 4:**
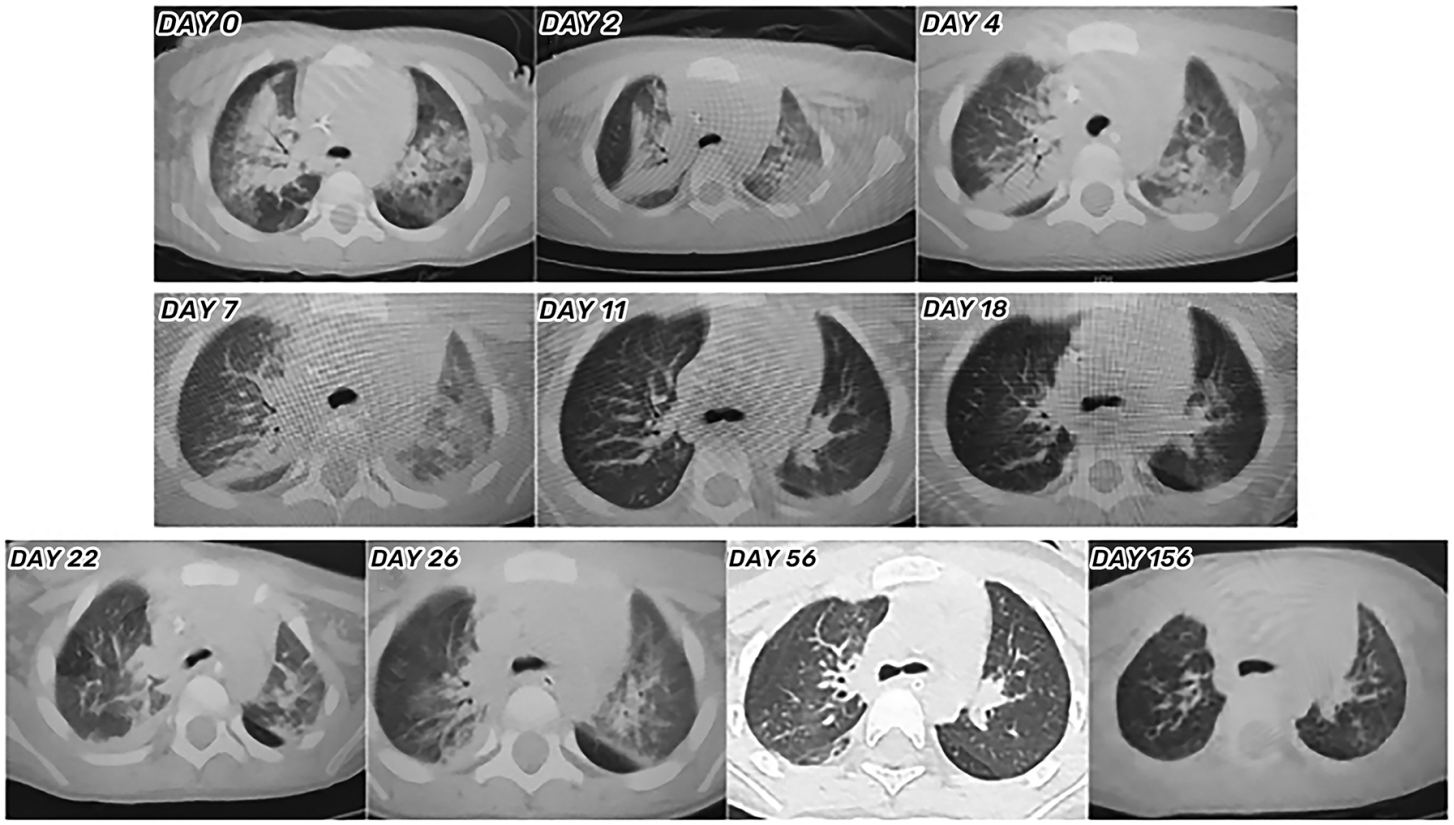
Lung computed tomography scans of the toddler during hospitalization and follow-up.

**Figure 5:**
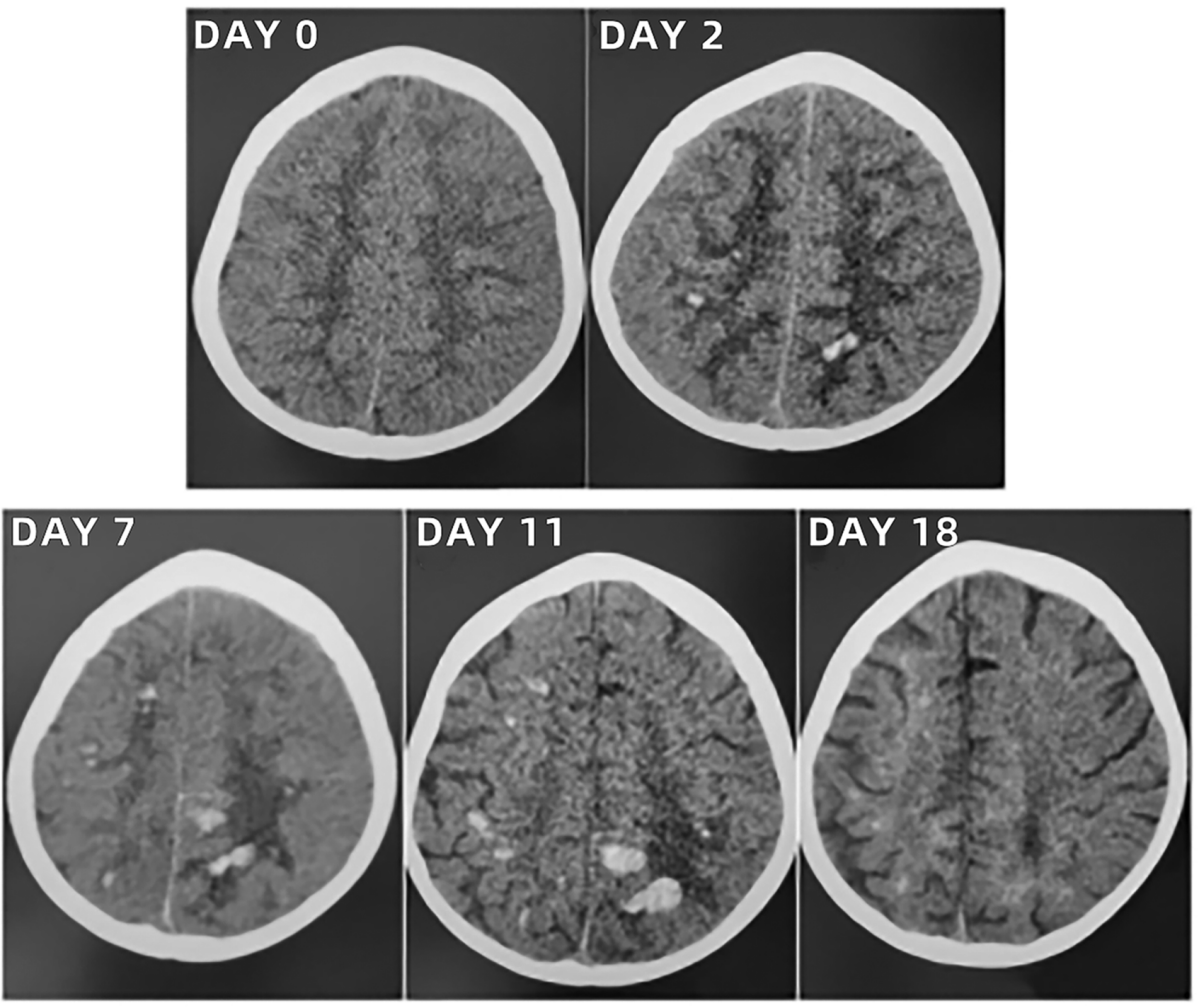
Brain computed tomography scans of the toddler during hospitalization.

**Figure 6:**
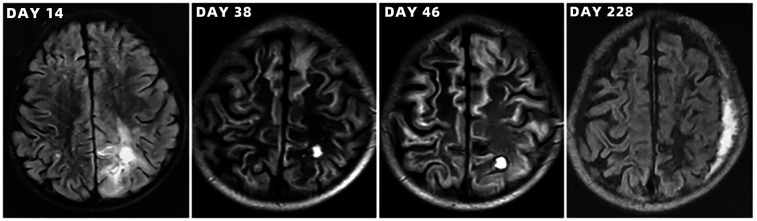
Brain magnetic resonance imaging scans of the toddler during hospitalization and follow-up.

The initial lung computed tomography (CT) scan revealed scattered, patchy shadows in both lungs with indistinct boundaries and visible air-bronchograms. Echocardiography (ECHO) indicated minor regurgitation of the mitral and tricuspid valves, along with a low limit of left ventricular systolic function (LVSF). Abdominal ultrasound (US) displayed upper abdominal bloating, diffuse lesions in both kidneys, and elevated resistance in blood flow (BF) of both renal arteries (RA). Other imagings upon admission, including brain CT scan and US, and chest and lower limbs vascular US, showed no remarkable findings. Imaging findings upon admission and subsequent re-examinations during hospitalization and follow-up were displayed in Figures 4–6.

### Diagnosis upon admission

The toddler was diagnosed as severe CAP, septic shock, and multiple organ dysfunction syndrome (MODS) based on the history of present illness, clinical presentations, physical examination, laboratory parameters as well as imaging findings. However, the etiology of recurrent convulsions had not been identified at that point.

### Treatment

After admission to ICU, comprehensive therapies, including appropriate analgesia and sedation, orotracheal intubation, invasive mechanical ventilation (IMV) with lung-protective ventilation strategies, prone position, goal-directed fluid resuscitation, infusion of vasoactive and positive inotropic agents, empirical selection of ceftriaxone sodium as the initial antibiotic, application of corticosteroids to mitigate inflammation-induced multiple organs damage, immunoglobulin (Ig) pulse (400 mg/kg) to enhance immunity, levetiracetam for anti-epileptic therapy, dehydration for brain protection, protection of multiple organs function, and administration of H_2_ receptor antagonists to prevent stress ulcers, were implemented. Flexible fiberoptic bronchoscopy (FFB) revealed a moderate amount of yellow mucus sputum in the airway with congestion and swelling of the airway mucosa. Subsequently, bronchoalveolar lavage (BAL) was performed to collect samples for mNGS. On the second day after admission, mNGS of bronchoalveolar lavage fluid (BALF) identified the pathogen as SA with the highest sequence number of 10,954 and natural resistance to aztreonam, polymyxin B/colistin, and nalidixic acid. Due to a sharp increase in serum creatinine (SCr) to 311.00 ummol/L and a rapid decrease in platelet (PLT) to 94 × 10^9^/L, in the dilemma of vancomycin and linezolid, we chose the latter combined with ceftriaxone sodium for anti-infective therapy. A repeat ECHO showed that cardiac function had returned to normal. However, it was important to note that the re-examination of brain CT scan revealed multiple intracerebral hemorrhage (ICH) and ischemic changes in the white matter behind the bilateral lateral ventricles, the etiology of which needed to be further clarified after laboratory parameters approached normal levels. On the fourth day after admission, the re-examination of lung CT scan indicated no significant change in the extent and severity of bilateral pneumonia compared to the initial assessment. However, PLT had decreased to 30 × 10^9^/L, and coagulation and kidney function had recovered somewhat but remained noticeably abnormal, which seriously undermined our confidence in continuing to use linezolid or switching to vancomycin. The continued decline in PLT leading to an increase in ICH or the deterioration of kidney function requiring continuous renal replacement therapy (CRRT) was too much for this toddler. After careful consideration and obtaining informed consent from the legal guardian of the toddler, given that enteral nutrition (EN) had been reestablished, compassionate use of contezolid (200 mg twice daily) was chosen in place of linezolid to avoid potential ADRs associated with vancomycin and linezolid. On the 12th day after admission, the toddler was successfully weaned from IMV and transitioned to mask oxygen inhalation following cuff leak test (CLT) and 30-minute spontaneous breathing trial (SBT). With PLT and coagulation function nearly returning to normal, lumbar puncture was performed for laboratory examinations of cerebrospinal fluid (CSF), including mNGS, microbial cultivation, routine analysis, biochemical detection, Ig determination, and acid-fast bacillus testing, all of which came back negative except for elevated IgA and α2-macroglobulin. After ruling out the possibility of central nervous system (CNS) infection, ceftriaxone sodium was replaced with cefoperazone/sulbactam sodium. On the 13th day after admission, the toddler underwent a tracheotomy procedure to maintain airway patency and prevent the accumulation of airway secretions and recurrence of lung infection, as she remained in a comatose state with occasional convulsions and limited self-cleaning ability of the airway. On the 14th day after admission, brain magnetic resonance imaging (MRI) scan revealed multiple ICH in bilateral cerebral parenchyma, primarily located in the left parieto-occipital lobe and in the early stage of sub-acute phase, with no abnormalities detected in intracranial vessels (Figure 3). On the 19th day after admission, contezolid was discontinued. On the 22nd day after admission, the toddler was stable and transferred to the pediatric ward, but remained unconscious with children GCS score of 11 points and occasional convulsions, manifested as limb stiffness.

### Outcome and follow-up

On the 30th day after admission, the toddler was discharged and transferred to the rehabilitation hospital for further neurological rehabilitation, with children GCS score of 13 points. Follow-up CT and MRI scans of the toddler are shown in Figures 4–6. The last follow-up lung CT scan on the 126th day after discharge showed bronchitis with localized inflammation. The last follow-up brain MRI scan on the 199th day after discharge revealed left parietal subdural hematoma, cerebral atrophy, and multiple encephalomalacia foci. At the last follow-up on the 280th day after discharge, it was noted that the toddler had regained consciousness, presented as completing simple actions on command, pronouncing a single sound and standing up with support from a chair, except for poor hand-eye coordination and inability to roll over independently.

## Discussion

CAP, usually considered as a major global health issue, represents a leading cause of hospitalization, large consumption of antibiotics, need for ICU-level care and organ function support, and substantial mortality in pediatric population younger than 5 years, thus posing a significant threat to childhood health, placing a huge burden on healthcare systems, and presenting a grand challenge to clinical management (7, 22, 23). As a common acute lower respiratory tract infection, pediatric CAP is characterized by a predominance of respiratory viruses over other pathogens, with RSV being the most frequently isolate, especially in pediatric population under 2 years of age (24, 25). However, the etiologies and prevalences of CAP vary substantially depending on the age breakdowns, individual susceptibilities, clinical settings, geographical regions, seasons and epidemiological characteristics (26, 27). For instance, in Saudi Arabia, SA, a Gram-positive bacterium, has been identified as one of the major contributors to pediatric CAP (28). Additionally, SA is a frequent pathogen of bloodstream infection (BSI), which can worsen the condition in children with CAP (29, 30). Finally, local or systemic complications, acute respiratory distress syndrome (ARDS), disseminated intravascular coagulation (DIC), multiple organ involvement or even dysfunction often serve as crucial clinical indicators of disease deterioration and poor prognosis in children with CAP, and SA is one of the common culprits (31).

SA can be categorized into methicillin-susceptible SA (MSSA) and MRSA based on the presence or absence of methicillin-resistance, with panton-valentine leukocidin (PVL) being one of the most important virulence factors to cause leukocyte lysis and tissue necrosis in clinical settings (12, 32). Consequently, SA is an extreme-virulent and high-pathogenic Gram-positive bacterium and capable of rapidly developing antibiotic resistance (33). Furthermore, the prevalences of SA-induced a series of diseases have shown a steadily increasing trend over the years due to the introduction of pneumococcal conjugate vaccines (PCVs) (34, 35). Prompt and accurate pathogen identification provides objective scientific evidences for optimal amtimicrobial stewardship strategies to reduce excessive use of antibiotics, secondary resistance of strains, potential ADRs and unnecessary costs. However, challenges such as insufficient awareness of pathogen detection, limited access to pathogen acquisition, low positive yield and relative hysteresis of traditional detection methods, and uncertainty of detection results significantly impede the timely and precise management of CAP in clinical practice (36). Rapid, highly accurate, readily available, and cost-effective microbiological diagnosis for the etiologies of CAP may be a breakthrough for this predicament. With its extensive clinical application, mNGS has gradually demonstrated greater potential than traditional detection methods for early pathogen identification to facilitate rational use of antibiotics, especially in BALF samples from patients undergoing IMV (37–39).

In this toddler, mNGS of BALF sample collected immediately after IMV revealed the culprit was SA with natural resistance to aztreonam, polymyxin B/colistin and nalidixic acid. Despite the lack of detection of methicillin-resistance, first-line anti-MRSA antimicrobial agents were chosen for salvage therapy in view of disease severity, progress in lung imagings, and multi-organ involvement of the toddler upon admission. Even if the responsible pathogens are identified, antibiotic regimens must take into account some specific clinical characteristics such as pharmacokinetics (PK), pharmacodynamics (PD) and potential ADRs associated with the selected antibiotics, disease severity, immune status, organ function and economic affordability of patients, and history of antibiotic use (40). More importantly, failure in initial empirical antimicrobial therapy can be associated with unfavorable outocmes (41). The decision to off-label use of contezolid was driven by concern about potential ADRs associated with vancomycin and linezolid, which could further make the already serious condition worse (18). Although contezolid is officially approved for managing cSSTI induced by SA, streptococcus pyogenes or streptococcus agalactis, but in fact, it has demonstrated broad-spectrum and potent activity against Gram-positive bacteria from various parts of the body, including multidrug-resistant (MDR) strains, in both in-vivo and in-vitro studies with an improved safety and tolerability profile through minimizing effects associated with myelosuppression and monoamine oxidase (MAO) inhibition (18, 19, 42–45). As the only completely synthetic class of antibiotic, contezolid exerts a unique mechanism of action by inhibiting targeted protein synthesis and exhibits excellent dissemination to lung tissues (46). After continuous oral administration of contezolid (200 mg twice daily) for 15 days on ordinary fed status, the toddler achieved the expected therapeutic effect and favorable outcome without any ADRs. In addition, high CSF permeability of unbound contezolid has been demonstrated in a patient with tuberculous meningoencephalitis, suggesting the therapeutic potential of contezolid for CNS infections (47).

Another noteworthy issue in this case was the occurrence of secondary multiple ICH and ischemic changes in the white matter behind the bilateral lateral ventricles following severe CAP, which was really a rare phenomenon in clinical practice. These near-complete negative CSF tests dispelled our initial suspicions of a CNS infection. During the subsequent multidisciplinary team (MDT) discussion, the majority consensus was that these neurological abnormalities were primarily linked to severe CAP-induced MODS, especially coagulation dysfunction. Insufficient evidence existed to suggest the involvement of secondary cytokine storm in the brain. An open question for consideration was whether 12-day systemic antibiotic therapy in conjunction with 5-day Ig pulse could eliminate the pathogens responsible for these neurological abnormalities, even though we were not inclined to favor this possibility. The last follow-up lung CT scan on the 126th day after discharge indicated bronchitis with localized inflammation, suggesting that it might take longer to repair SA-caused severe lung damage in the toddler. The results of the last brain MRI scan and neurological follow-up revealed the persistent severity of brain injury and sequelae in the toddler with SA-induced severe CAP, which could have a substantial impact on the growth and development of children.

A limitiation of this study is the lack of objective conditions for monitoring PK and PD of contezolid, which is particularly important when using newly developed drugs in pediatric patients, especially for off-label use. In addition, the successful application of contezolid in the management of SA-induced severe CAP in this toddler is only a case report, which is not universal, and can not be directly extended to similar patient populations or indications. Lastly, although this toddler was followed up to 280 days after discharge, it was still insufficient.

## Conclusion

Although contezolid has not been officially indicated for CAP, it has been shown to be effective and safe in the management of SA-induced severe CAP in this toddler, suggesting its potential as an alternative option in the dilemma, especially for patients who are susceptible or intolerant to ADRs associated with first-line anti-MRSA antimicrobial agents. Future efforts should concentrate on confirming our findings through more similar cases and well-designed clinical trials.

## Data Availability

The original contributions presented in the study are included in the article/Supplementary Material, further inquiries can be directed to the corresponding authors.
