# Supplementary figures and images for: Compassionate use of contezolid in a toddler with severe community-acquired pneumonia induced by staphylococcus aureus: a case report and follow-up
Source: Front Pediatr. 2024 Feb 7;12:1321447. doi: 10.3389/fped.2024.1321447 (PMC10879426; doi:10.3389/fped.2024.1321447)

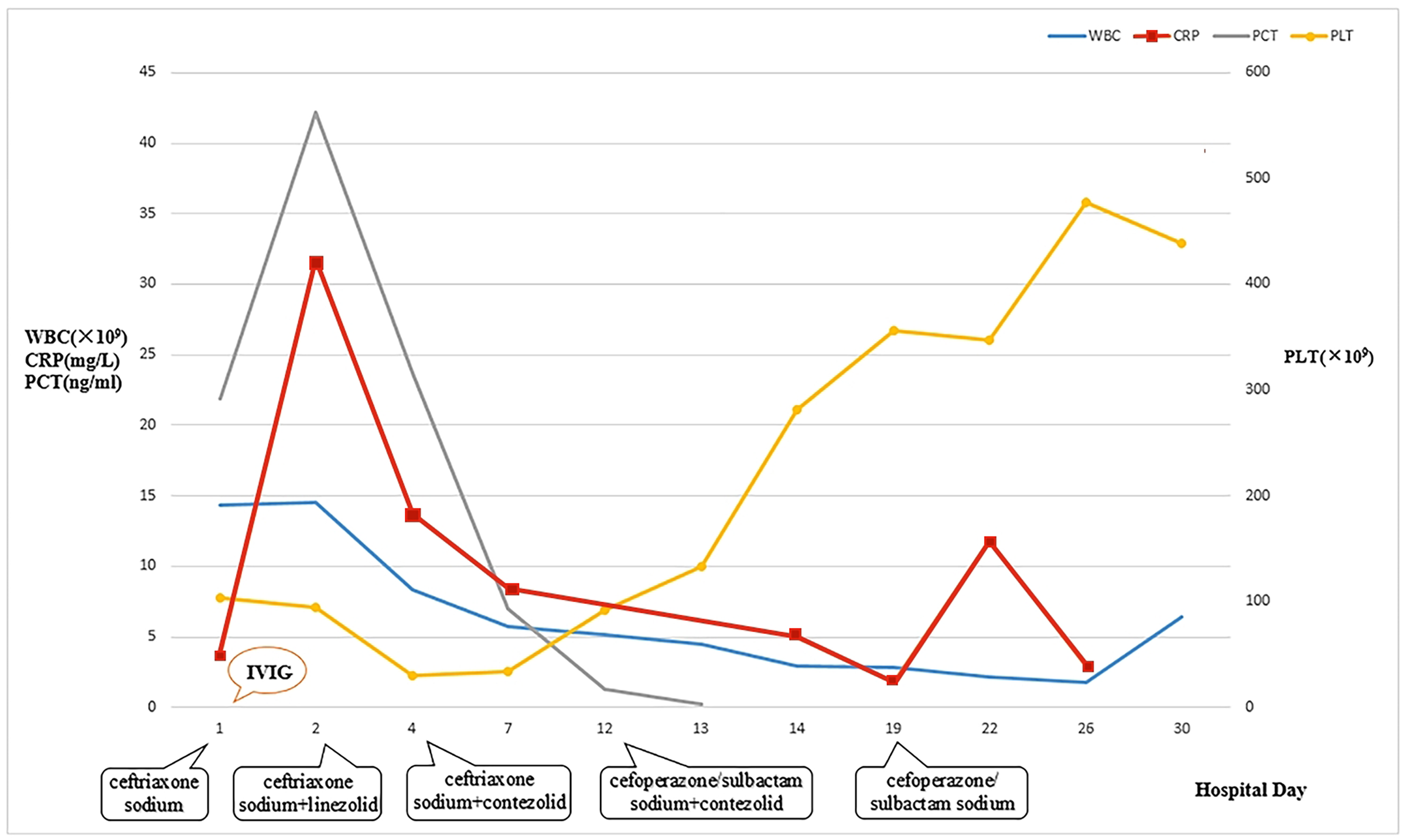

Supplement: Supplementary file 1 [file Image1.tif]

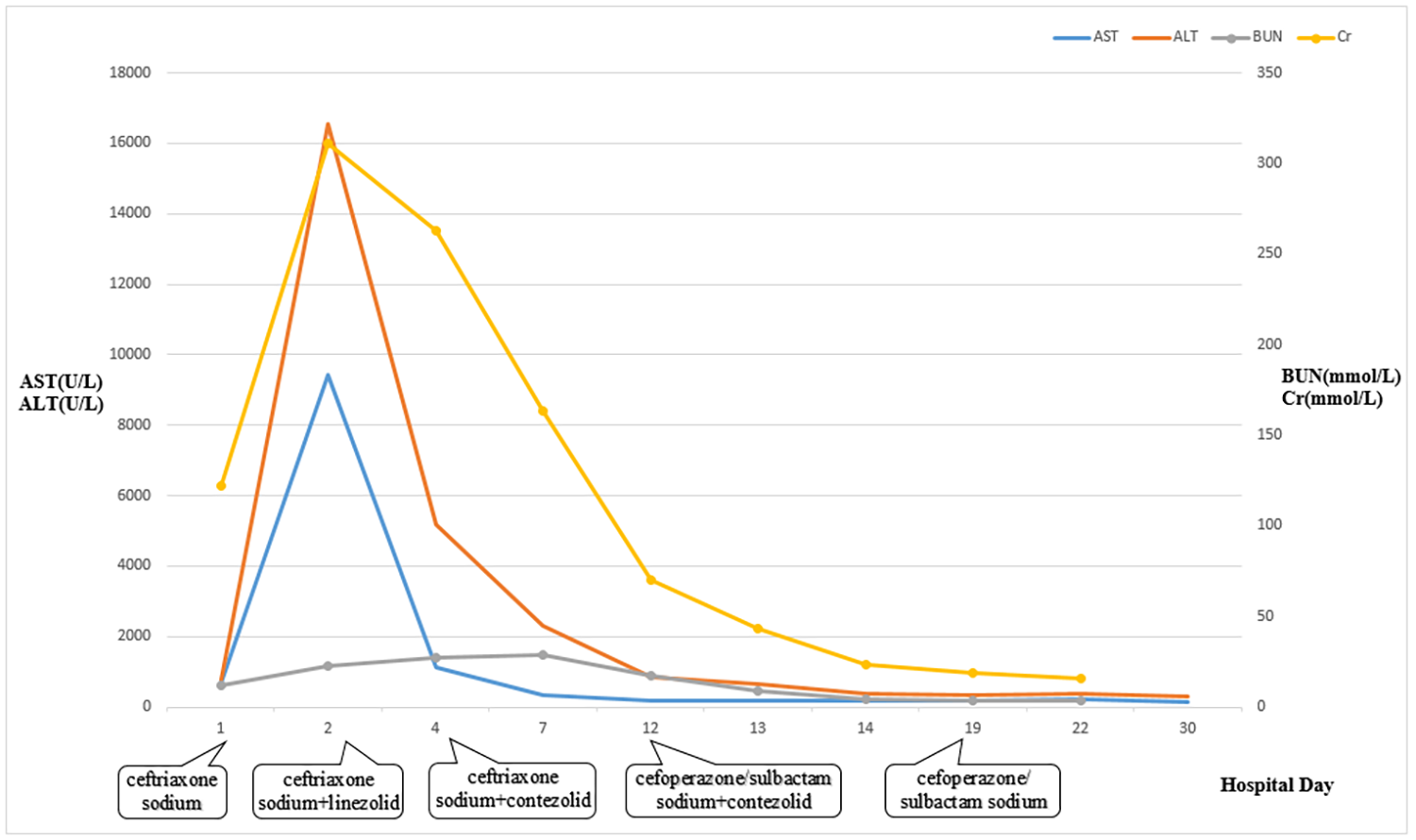

Supplement: Supplementary file 2 [file Image2.tif]

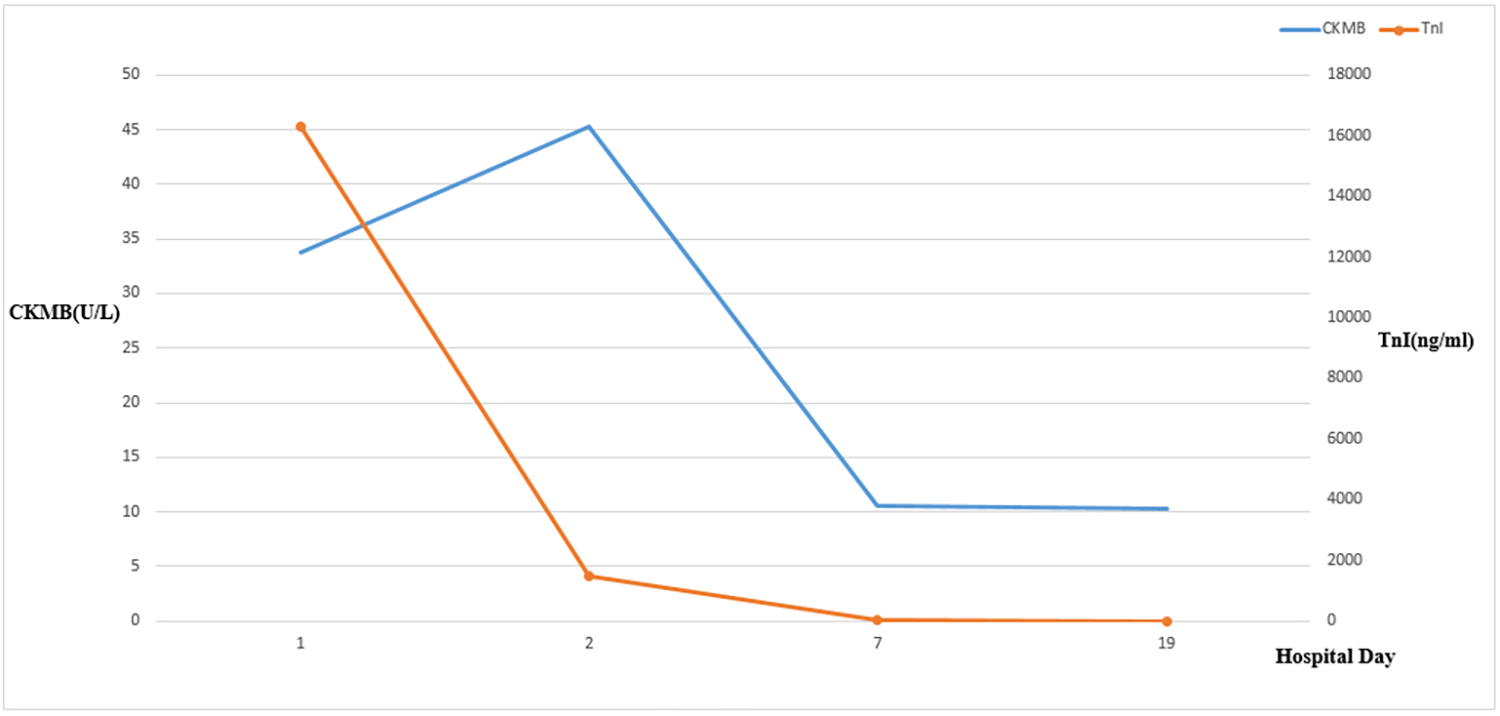

Supplement: Supplementary file 3 [file Image3.tif]
